# Can body condition and other external characteristics predict semen production and quality in layer roosters?

**DOI:** 10.1016/j.psj.2026.107697

**Published:** 2026-08-25

**Authors:** Eder B. Oliveira, Edvaldo F.S. Fogaça, Alan M. Durigon, Marcílio Nichi, Ricardo J.G. Pereira

**Affiliations:** a MSD Saúde Animal, São Paulo, SP, Brazil; b Mercoaves Comercio de Aves Ltda, Tietê, SP, Brazil; c Laboratory of Andrology, Department of Animal Reproduction, College of Veterinary Medicine and Animal Sciences, University of São Paulo, Av. Duque de Caxias Norte 255, CEP 13635-900, Pirassununga, São Paulo, SP, Brazil; d Study Group for Avian Multiplication – GEMA, Department of Animal Reproduction, College of Veterinary Medicine and Animal Sciences, University of São Paulo, Pirassununga, Brazil

**Keywords:** Layer, Breeders, Phenotype, Selection, Reproductive management

## Abstract

Male fertility is one of the main factors determining the reproductive efficiency of a breeding flock, and aging is a process that impacts the production of fertile eggs in both broiler and layer lines. With the aim of developing selection criteria for males, phenotypic aspects are routinely used in the rearing and production phases allowing, for example, broiler males to reach puberty at the appropriate age without delays in production, premature decline, or low fertility persistence. However, layer lines were not selected for the same traits over the decades, and consequently, the roosters are lighter, more agile, and do not exhibit problems related to body conformation, pododermatitis, or abdominal fat accumulation as their broiler congeners. Therefore, we question whether the phenotypic aspects routinely assessed in layer lines are as useful in predicting semen production and quality as in broiler lines. Thus, 50 Bovans White roosters from a commercial parent-stock house were periodically monitored from 26 to 86 weeks of age for their body characteristics and semen parameters. Aging had a significant effect on the number of progressive spermatozoa, but individuals of different weight or fleshing categories showed no differences in this variable. Males with below-standard weights exhibited higher percentages of inactive cloacas and unsuccessful semen collection than those with standard or above-standard weights. We also observed that roosters with lower fleshing scores had higher percentages of inactive cloacas but no difference in semen collection success rates. Although the success rate for semen collection was higher in roosters with active cloacas, no differences in the number of progressive spermatozoa was found between individuals with active and inactive cloacas. Taken together, these data expand our understanding of the applicability of selecting layer roosters based on weight and fleshing. Further research regarding the correlation between these indicators and steroidogenic activity, sexual behavior and, above all, fertility in layer lines is needed. This information can greatly contribute to the processes of genetic improvement and reproductive management of layer lines.

## Introduction

Over the years, new technologies have been introduced to further enhance the genetic development of breeding lines, and the analysis of the phenotype of parent flocks is one of the most widely used tools as it reflects the interaction between genotype and environment (Hy-line, 2021; Hendrix-Genetics, 2022; Lohmann, 2024). The rearing period is of great importance for the performance of breeding stocks as, in general terms, the productivity of a flock depends on achieving body weight targets from an early age. During the first 5 weeks of life, for example, the main objectives of rearing are: to ensure rapid growth in order to achieve an adequate minimum weight, to maintain good flock uniformity, and to reach a high postnatal survival rate (Hy-line, 2021; Hendrix-Genetics, 2022; Lohmann, 2024). Such care at this stage will guarantee the proper development of the immune system, vital organs, and body structure. Any delays during this period result in negative impacts on body composition and, consequently, on the future performance of the breeding stock.

It is well known in the poultry industry that the ability of parent flocks to reach their full reproductive potential depends on the synergy between factors such as nutrition, biosecurity, welfare, lighting and management (Macari, 2018). In recent years, considerable attention has been paid to the management of breeding roosters, given their relevance to issues of growing global concern, such as fertility and hatching rates (Cash et al., 2025). For this reason, genetic companies have been refining their male monitoring and selection processes over time. In general, they are all based on the assessment of phenotypic traits due to their applicability and presumed correlation with certain reproductive parameters. In broiler males, for example, optimal performance occurs when individuals gain weight continuously but in a controlled manner, both during the rearing and production phases (Macari, 2018). This rigorous weight control must take into account aspects as the proliferation of the different testicular cells and the mobilization of body reserves. Otherwise, roosters will be unable to maintain high rates of sexual activity and fertility (Brake, 1998; Vizcarra et al., 2010, 2015). Broiler males that are underweight during rearing may exhibit lower fleshing scores that hinder copulation, occupy lower hierarchical positions, and either delay or prematurely decrease their steroidogenic and spermatogenic activities (Sarabia Fragoso et al., 2013; Oviedo-Rondón and Romero-Sanchez, 2022). Conversely, being overweight can negatively affect fertility due to issues linked to body conformation (posture, fleshing, and cloaca), mobility (leg deformities and pododermatitis), or libido (accelerated testicular regression) (Hocking and Bernard, 1997; Robinson et al., 2003; Rezende et al., 2014; van Emous, 2019).

Weight checks and assessments of other phenotypical traits are also part of the routine in parent, grandparent and great-grandparent stocks of layer lines, especially during the rearing phase. The main objective is to ensure the proper development of the various systems, flock uniformity, and consequently, the breeders’ performance during production phase. However, for decades, intensive genetic selection for broilers and layers has resulted in profound differences among lines regarding feed intake, growth rate, nutrient utilization efficiency, and development of muscles and adipose tissue (Buzala and Janicki, 2016). Furthermore, improvements in reproductive traits and most management practices in layer lines have focused more on females than on males, due to the ease of monitoring their reproductive performance (Leenstra et al., 2016; Romero-Sanchez et al., 2025). Together, these particularities led us to question whether evaluation of physical characteristics such as body weight, fleshing score, comb, wattle and cloaca in males of layer lines is equally applicable in predicting the semen quality as it is in broiler males. Thus, with the aim of investigating the relationship between physical characteristics and semen parameters, this study monitored 50 Bovans White roosters kept under natural mating at a commercial parent stock house throughout their productive cycle (26 to 86 weeks of age). We believe that our findings can contribute to improving current male selection processes at breeding farms during the rearing and production periods, positively impacting the layer industry in Brazil and worldwide.

## Material and methods

### Experimental design

All procedures complied with the current regulations established by the Ethics Committee of the College of Veterinary Medicine and Animal Sciences at the São Paulo University (CEUAVET No 5477171122). This study was conducted with 50 randomly selected Bovans White roosters, which were marked and kept with other males and females in a commercial parent stock house under standard environmental, management, lighting and nutritional conditions established by Mercoaves Comércio de Aves Ltda. All individuals were assessed at approximately 10-week intervals from 26 to 86 weeks of age, during which information was collected on their semen quality and general condition (body weight, fleshing score, feet condition, comb and wattle color, shank and beak color, and cloaca appearance). Each ejaculate was individually analyzed for volume, concentration and total and progressive motilities.

### Flock management

Throughout the production phase, males and females were kept on the floor on a bed of wood shavings which was only replaced during the sanitary break. Both sexes fed from the chain feeder systems (10 cm per bird) with feed being released four times a day (118 grams per bird). Chlorinated water (5 ppm) was provided *ad libitum* via nipple drinkers. Lighting was maintained at 16 h of light per day (from 4:00 am to 8:00 pm) in an open-sided house equipped with yellow side-curtains, and the intensity of artificial light during dark periods was kept at 60 lux. The internal temperature in the house was set at 25°C, which was maintained by fans spaced 15 meters apart. The density of birds was 8 birds per square meter, and the density of manual nests was 4 birds per nest.

### Procedures prior to semen collection

The parent stock house comprised 1,700 and 16,000 Bovans White roosters and hens, respectively, which distributed among 4 identical compartments. Inside two of these compartments, we built 2 pens (3 × 3 meters) with waterers and feeders to separate the marked males during the collection periods (25 individuals per pen). Three days before each collection, males were kept in these pens with water and food *ad libitum* to prevent access to females and to perform cloacal feather trimming. This separation procedure was necessary to ensure standardization of the semen collections and to obtain sufficient ejaculate volumes for analysis. Semen collection and analysis were performed inside the parent stock house within 10 min to minimize any variations related to storage or transport.

### Semen collection and analysis

Semen was collected from roosters using the dorso-abdominal massage method (Burrows and Quinn, 1937), and all ejaculates were assessed macroscopically and microscopically by the same investigator. The macroscopic parameters considered were volume, color, appearance, and contamination of each ejaculate. Volume was measured in milliliters (mL) using variable volume pipettes. Ejaculates that exhibited a greenish or reddish color were discarded due to contamination with feces and blood, respectively. Sperm concentration of clean ejaculates (i.e., without feces or urine contamination) was immediately determined by spectrophotometry (Accuread rooster and turkey photometer, IMV, France), and final concentration (100 × 10^6^ spermatozoa/ mL) was achieved by diluting each sample with Biggers-Whitten-Whittingham medium (Blank et al., 2021). Total and progressive motility were assessed subjectively using light microscopy (Nikon® E200, Nikon, Tokyo, Japan). Total motility was calculated as a percentage (0 – 100%) taking into account the proportion of moving sperm, whereas progressive motility considered only the proportion of sperm moving forward in a straight line (0 – 100%) (Blank et al., 2021). These motility parameters were estimated after evaluating at least 10 different fields from each sample. Subsequently, measurements of volume, concentration and progressive motility were used to calculate total progressive sperm per ejaculate (*TPS= V x C x [P/100]*, where V is seminal volume in mL, C is sperm concentration in billion per mL, and P is progressive motility as a percentage). This index was used in all statistical analyses because it conceptually represents the viable spermatozoa that have the actual ability to reach the females’ sperm storage glands after copulation.

### Assessment of the physical characteristics of breeding males

After each semen collection, males were weighed and evaluated for their body and reproductive traits. Each individual’s weight was compared to the standard range established by the Bovans White parent stock product guide for the age in question, and then roosters were categorized within each collection period as (AS) above standard, (S) standard, or (BS) below standard. Fleshing scores were: (H) high, keel very well-muscled with the tip of the keel difficult to palpate; (G) good, keel adequately filled by muscles (in a ‘V’ shape) without difficulty in palpation; (L) low, prominent keel with little musculature; (+Le) keel with lesions; and (Cr) crooked keel. Four categories were established for foot condition: (N) normal, with no swelling, sores or redness; (P) pododermatitis, severely swollen, prominent sores or scabs; (CT) crooked toes; and (MP) missing phalanges. Comb and wattles were classified according to their color: (N) normal (red); (Y) Yellowish; (B) Bluish (cyanotic); and (W) whitish (pale). Three scores were assigned for tarsus and beaks: (N) normal (very yellowish); (W) whitish (pale) and (B) bluish (cyanotic). Regarding the roosters’ physical, we listed two possible situations: (N) normal (active/aggressive); and (L) lethargic (apathetic/ sluggish). Finally, the cloaca was classified as: (A) active, open, moist and pinkish in color; and (I) Inactive, small, dry, and pale.

### Statistical analysis

All statistical analyses were performed using the Statistical Analysis System 9.4 software (SAS Institute, Cary, NC). Variables were initially tested to determine variance homogeneity and residual data normality by PROC GLM (Bartlett’s test) and PROC UNIVARIATE (Shapiro-Wilk test). Since these assumptions were not met even after applying different types of data transformation, nonparametric analyses were used for statistical comparisons. The experimental unit was either the ejaculates or the individual depending on the comparison being made. The effects of the variables (e.g. weight category, weight tertile, fleshing score, and cloacal condition) and the age of the flock (in weeks), as well as the interaction between these factors, were estimated using repeated measure analysis of variance (SAS Mixed Procedure). Next, comparisons between the means of two groups were performed using Wilcoxon test, whereas Kruskal-Wallis test was used for comparisons among three or more independent groups (followed by Dunn’s multiple comparison test when necessary). Only total progressive spermatozoa per ejaculate (TPS) was used in all comparisons related to sperm production, since this index represents the viable cells that effectively reach the females’ sperm storage glands following copulation. All data are expressed as mean ± SEM, with statistical significance defined as P < 0.05. Additionally, chi-square tests of independence were conducted to examine the relationship between: weight category and cloacal condition; weight category and semen collection success; fleshing scores and cloacal condition; fleshing scores and semen collection success; and cloacal condition and semen collection success. When significant relationships were found (p < 0.05), we performed Pearson’s residual analysis. Residual values greater than 1.96 (positive or negative) indicate that the observed frequencies is significantly different form the expected frequency, contributing to the rejection of the null hypothesis of independence.

## Results

The general data regarding body weight and weight category percentages (according to the parent stock guide), fleshing scores, cloacal condition, and semen collection success of Bovans White roosters evaluated between 26 and 86 weeks of age are summarized in Fig. 1. Based on this information, we observed that individuals at the beginning of the production phase (26 weeks of age) exhibited average weight below the standard, but from 49 weeks onward they remained well above the standard range (Fig. 1A). It was also possible to notice that weight variation at 26 and 35 weeks of age was around 420 grams (coefficients of variation – CVs – of 5.98% and 5.26%, respectively), but after 49 weeks, it reached differences between 660 and 915 grams (CVs between 6.26% and 7.86%). Such a significant increase in weight after 49 weeks of age was clearly observed when we categorized roosters according to the standard weight range described in the line guide, where more than 82% of the males weighed above the standard (Fig. 1B). The highest percentages of males within the standard range were found at 26 and 36 weeks of age (10 and 12%, respectively), whereas in the other age groups the percentages with standard weight ranged from 0% to 4%.

**Fig. 1:**
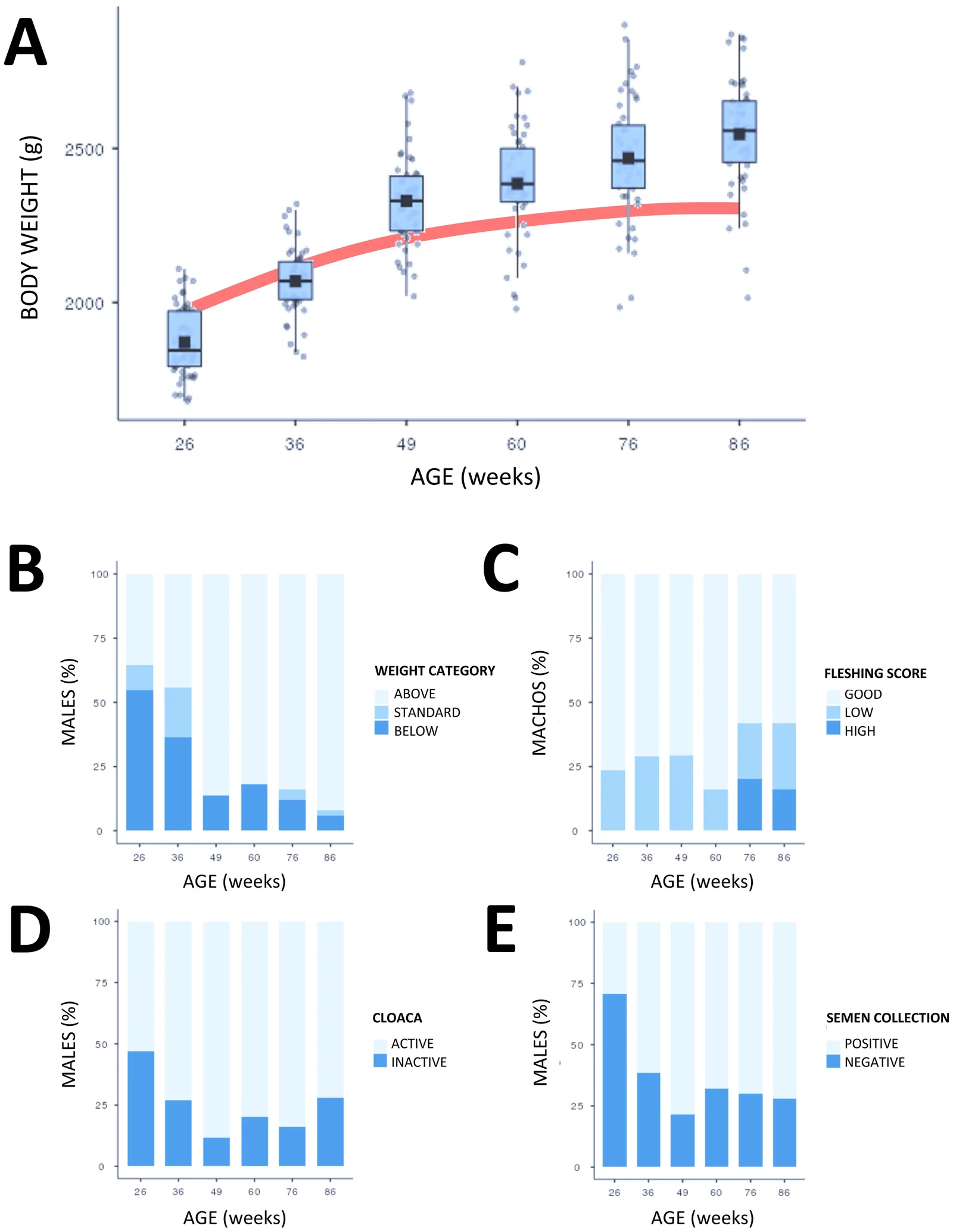
Data from Bovans White roosters housed in a commercial parent stock house and monitored from 26 to 86 weeks of age. (A) Box-plot illustrating the distribution of body weights of the collected roosters in relation to the weight range established by the parent stock product guide (red line, where the thickness covers the interval between the minimum and maximum weights to be considered within the standard); (B) Percentage of roosters in the above-standard, standard, and below-standard categories according to the parent stock product guide; (C) Percentage of roosters in the three breast scores defined by the company (good or “V”-shaped keel, low or prominent keel without significant musculature, and high or very well-muscled with a keel difficult to palpate); (D) Percentage of roosters exhibiting an active or inactive cloacas (i.e., individuals supposedly exhibiting copulatory behavior or not, respectively); and (E) Percentage of males where it was possible or not to obtain ejaculates after digital massage (positive and negative, respectively).

In parallel, the analysis of the fleshing scores demonstrated that most roosters throughout the monitoring period were classified as having good conformation, with the percentages with this category varying between 84% and 58% (at 60 and 76 weeks of age, respectively) (Fig. 1C). It is also worth noting that only from 76 weeks of age onwards did we detect some males with high fleshing scores. The lowest percentage of roosters with active cloacas (57%) was observed at the beginning of the production phase (25 weeks), whereas for the remainder of the study roosters showing active cloacas ranged from 72% to 88% (Fig. 1D). Similarly, the lowest success rate in semen collection occurred at 26 weeks of age (29%), while in the other periods we were able to collect ejaculates from 62% to 78% of the monitored males (Fig. 1E). Moreover, 99%, 94% and 100% of the evaluations carried out throughout the study classified the wattles and comb, tarsus and beaks, and feet as normal, respectively. These findings confirmed the good condition of the males regarding these physical traits, however, at the same time made any comparisons between average sperm production unfeasible due to the lack of representatives in the different categories.

Our statistical analysis did not reveal interactions between the effects of variables weight category, weight tertiles, fleshing scores and cloacal condition, and the effect of flock age on total progressive sperm per ejaculate (TPS) (Table 1), and therefore, the TPS results were grouped according to the independent variables (Fig. 2). Age exerted a significant effect on TPS (p < 0.05) with the minimum and maximum averages occurring at 26 and 40 weeks of age, respectively (Fig. 2A). Regarding weight categories following the line manual, no differences were ascertained between groups (p > 0.05); however, we verified a low number of data points in the standard range (n = 18) compared to the ranges above and below the standard (n = 214 and 72, respectively) (Fig. 2B). For this reason, in order to balance the number of observations per weight range, we simultaneously compared the TPSs among the weight tertiles within each age group (Fig. 2C). This approach also failed to demonstrate differences among the high, standard and low groups (p < 0.05). Likewise, fleshing score groups showed no differences in average TPS, although the number of individuals presenting high scores was quite low (n = 18) (Fig. 2D).

**Table 1:** Probability of interactions between the effects of the factors (weight categories, weight tertiles, flashing scores, and cloaca condition) and the effects of flock age (weeks) on the total progressive sperm per ejaculate.

Factor	Interaction effect	P
Weight category	Class x Age	0,6606
Weight tertile	Class x Age	0,5322
Fleshing score	Class x Age	0,8903
Cloaca condition	Class x Age	0,817

**Fig. 2:**
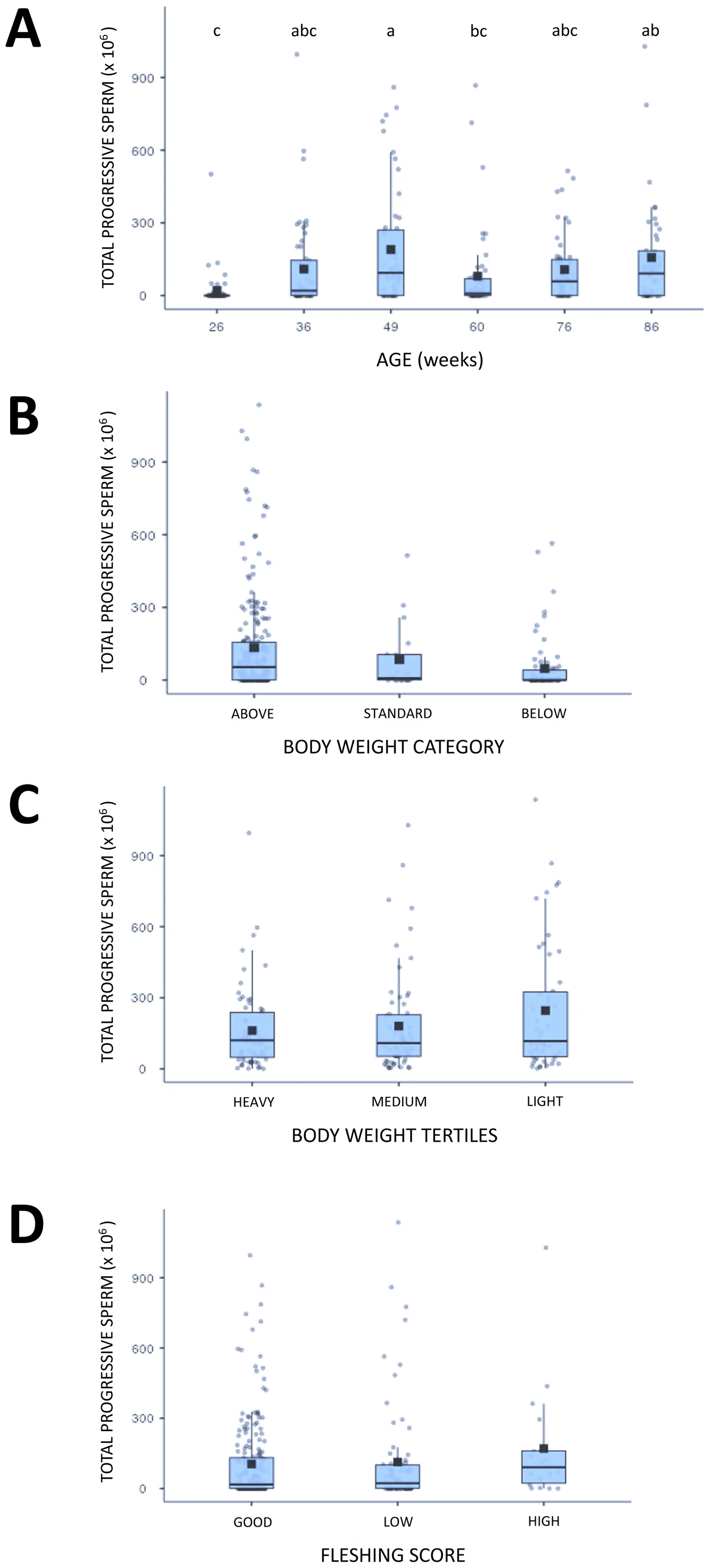
Total progressive sperm per ejaculate (TPS, in millions) of Bovans White roosters housed in a commercial parent stock house at different ages, weight categories, weight tertiles, and fleshing scores. (A) Box-plot of TPS values at the different ages included in our study. Different letters (a–c) indicate significant differences between ages (p < 0.05); (B) Box-plot of the number of TPS according to the weight category established by parent stock product guide (above standard, standard, and below standard); (C) Box-plot of the number of TPS according to the weight tertiles calculated within each age group (33.3% heaviest, 33.3% intermediate, and 33.3% lightest); and (D) Box plot of the number of TPS according to fleshing scores (good or “V”-shaped keel, low or prominent keel without much musculature, and high or very muscular with a keel difficult to palpate). There were no significant differences between groups in graphs (B), (C), and (D) (p > 0.05).

Comparisons of the percentages of males exhibiting active and inactive cloacas were also made across different weight categories and fleshing scores (Fig. 3). The chi-square test indicated a significant association between weight categories and cloacal condition, yet the observed frequencies differed from the expected frequencies only for inactive cloacas in the below-standard weight category (i.e., only this group appears to have a higher percentage of inactive cloacas, with no differences among individuals in the standard and above-standard weight categories) (Fig. 3A). Similar findings were perceived when studying the relationship between weight categories and the semen collection success, and between fleshing scores and cloacal condition (Fig. 3B and C). In contrast, no association was found between fleshing scores and semen collection success (Fig. 3D). Finally, we noted a strong relationship between cloacal condition and semen collection success (with the observation of active cloacas strongly associated with semen collection success) (Fig. 4A), nevertheless no significant differences were found regarding TPS production (Fig. 4B).

**Fig. 3:**
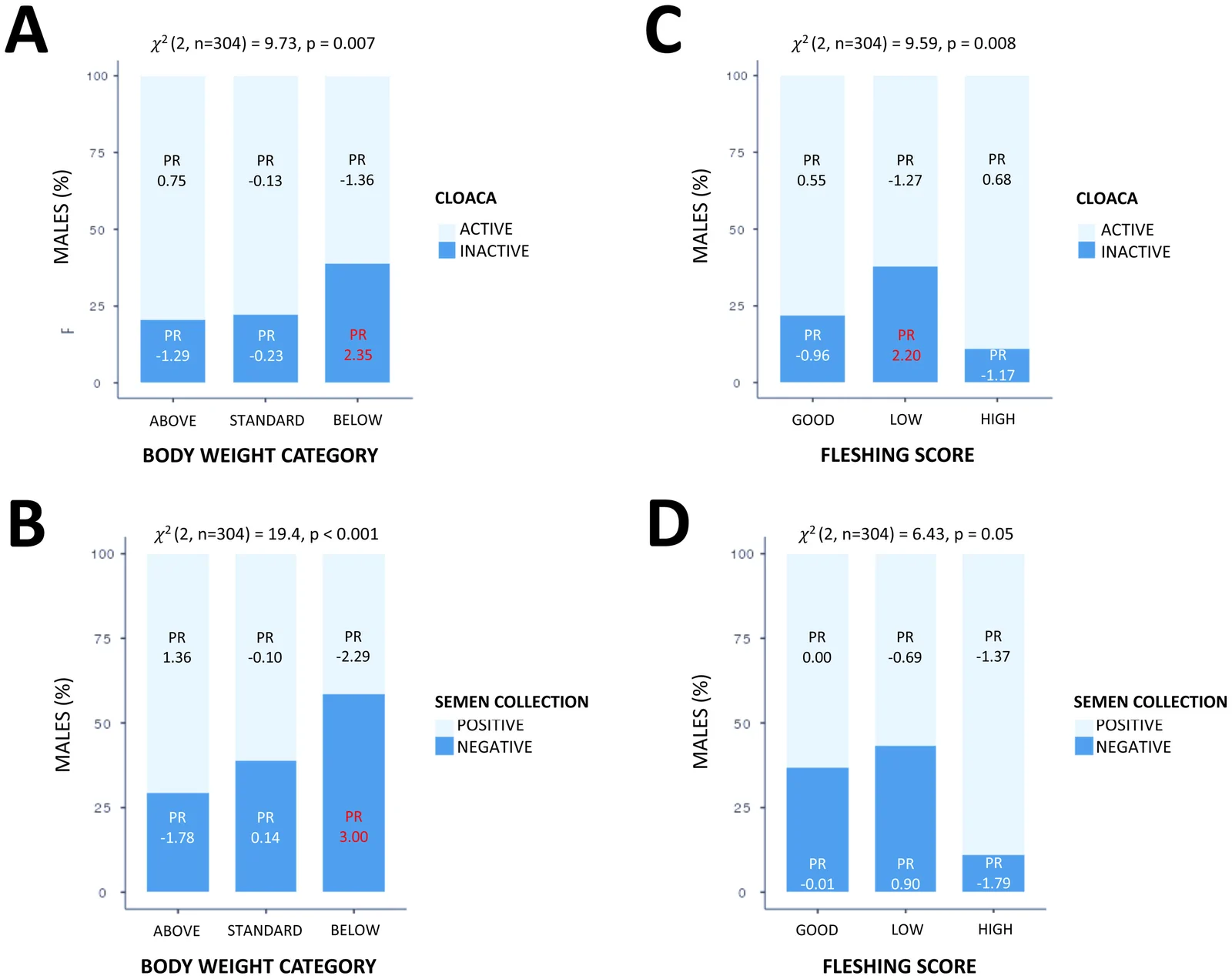
Frequencies of cloacal conditions and semen collection success rates in different weight categories and fleshing scores in Bovans White roosters housed in a commercial parent stock house and monitored from 26 to 86 weeks of age. (A, B) Percentages of males with active and inactive cloacas, and with successful or unsuccessful semen collection in the weight categories established by the parent stock product guide (above standard, standard, and below standard); (C, D) Percentages of males with active and inactive cloacas, and successful or unsuccessful semen collection across different fleshing scores (good or V-shaped keel, low or prominent keel without excessive musculature, and high or very muscular with a keel difficult to palpate). The detailed data above each graph refer to the results of the chi-square tests used to verify the associations between the two categorical variables. The values within each column indicate the PR (Pearson Residuals), and when these are greater than 1.96 or less than −1.96, they indicate a higher or lower frequency than expected, respectively (which, in this case, were indicated in red).

**Fig. 4:**
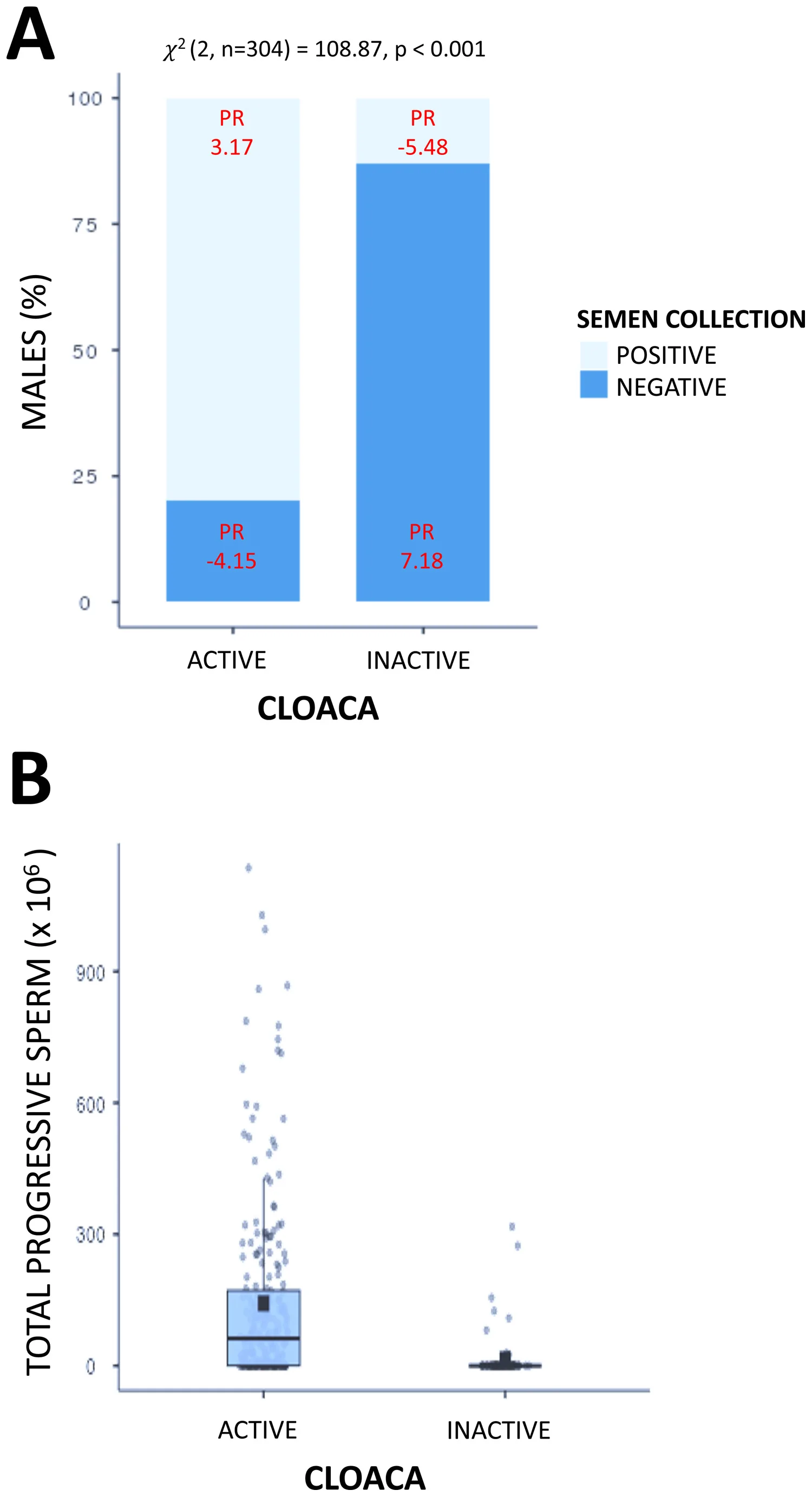
Frequencies of success rates in semen collection and total progressive sperm per ejaculate (TPS, in millions) according to cloacal condition in Bovans White roosters housed in a commercial parent stock house and monitored from 26 to 86 weeks of age. (A) Success rates in semen collection of males exhibiting active and inactive cloacas; and (B) Box-plot of TPS in males showing different cloacal conditions. The detailed data above each graph refer to the results of the chi-square tests used to verify the associations between the two categorical variables. The values within each column indicate the PR (Pearson Residuals), and when these are greater than 1.96 or less than −1.96, they indicate a higher or lower frequency than expected, respectively (which, in this case, were indicated in red). There was no significant difference between cloacal condition in graph (B) (p > 0.05).

## Discussion

The use of phenotypical traits in the selection process of breeding roosters is a routine procedure in parent, grandparent and great-grandparent flocks. It is a simple and immediate method to determine whether management and nutrition are providing the males adequate development and uniformity to ensure high fertility throughout their productive cycle. Yet, in the case of layer lines, parameters such as weight gain, feed intake, feed conversion, body conformation, among others, do not correspond to those observed in broiler lines. Therefore, we cannot expect changes in these characteristics to behave in the same way across distinct groups of poultry. In layer breeder operations, the fertility of a flock also depends heavily on the physical and reproductive fitness of the males. However, relying solely on body weight during the productive phase is not sufficient, as heavier males may simply have a larger skeletal frame rather than muscle and fat reserves. Because roosters are genetically lean, layer breeder companies tend not to use detailed fleshing scores as a primary selection tool, but they often palpate the keel to establish that birds are not emaciated or under conditioned. This ultimately helps those involved to assess males’ fitness, redefine feed allocations, and make culling decisions to maximize egg fertility.

Here, Bovans White roosters under commercial conditions (i.e., engaging in natural mating and social disputes with other males) were monitored throughout their production phase, not only in terms of physical traits but also regarding their semen parameters. Although these males at 26 weeks of age had an average weight below the line standard, they regained weight over time and achieved averages above the standard starting at 49 weeks of age (Fig. 1). This shift in weight pattern is generally observed because less attention is paid to layer males during the rearing phase as compared to females, followed by overfeeding to help these breeders to regain their weight. The situation was best illustrated by the weight category classifications, where after 36 weeks only a very small percentage of the males were identified as below or within the standard range. Such imbalance between categories was also observed for fleshing scores with the vast majority classified as good, and for traits such as the wattles, comb, tarsus and beak color, and the condition of the feet (over 94% in the same category). The homogeneity of the data confined our analyses solely to matters concerning semen production and weight, fleshing, and cloacal condition.

As expected, age was a factor that influenced not only the number of successful semen collection but also total progressive spermatozoa per ejaculate (TPS). With the exception of the collection at 26 weeks of age, the vast majority of roosters had active cloacas and positively responded to semen collection attempts. The lower initial performance is possibly linked to the birds’ maturation process, although the onset of sexual maturity at 26 weeks in Bovans White seemed somewhat late to us. This is because roosters from layer lines already show good fertility rates after 24 weeks of age (Hy-line, 2021; Hendrix-Genetics, 2022; Lohmann, 2024). Hence, assuming that body weight at the end of rearing is a good indicator of reproductive development, the high number of males with below-standard weight possibly explains the high percentage of inactive cloacas and failures in semen collection. Such assumption also substantiates the low TPS values revealed by our first semen analysis. However, we noticed that both the percentages of active cloacas and colletion successes, as well as the average TPSs showed a gradual improvement with age, peaking at 49 weeks of age.

Decreases in TPSs were recorded as the birds aged, but only the 60-week average was significantly lower than the peak at 49 weeks of age. Declines in fertility due to aging are a well-documented in broiler breeders, resulting from gonadal oxidative stress, dysfunction of the Sertoli and Leydig cells, and reduced synthesis and release of the FSH and LH, which ultimately lead to testicular regression (Weil et al., 1999; Avital-Cohen et al., 2013; Raei et al., 2021; Ansari, 2024). Unfortunately, the scarcity of information regarding semen production and quality in layer breeders across their reproductive cycle greatly limits more focused discussions on these lines. Nevertheless, the lack of differences between the peak at 49 weeks of age and the older ages may be the result of wide variations in TPS values within each age. This occurred because TPS calculation combines three seminal variables that, on their own, account for enormous interindividual variations. These variations also suggest that, despite many roosters were considered active based on cloacal inspection or successful semen collection, fewer birds actually produce good amounts of sperm to be transferred to the females. This hypothesis further reinforces the need for more efficient indicators to identify fertile males within the flock during routine operations. Another important point concerns the genetic selection process for layer lines, which for decades has prioritized reproductive traits to improve egg production (Buzala and Janicki, 2016). Therefore, it is not surprising that reductions in their semen parameters are less pronounced when compared to those described in broiler lines.

Considering that the weights of males naturally tend to increase with age, we chose to compare between weight categories in order to account for the age factor in our statistical analysis. Even so, no differences were found in terms of TPSs among males of the three weight categories. Notwithstanding, as mentioned earlier, we observed a significant inequality in the number of observations within each weight category with a lack of individuals in the standard weight range during certain periods. This scenario made a balanced analysis unlikely, because the high variability in TPS values requires multiple observations per weight category to detect differences between the groups. Thus, in an effort to equilibrate the number of observations per weight range, we also performed comparisons of TPSs among weight tertiles within each age group. (heavy, medium, and light). Similar to what was seen with weight categories, no differences in TPSs were found among the weight tertiles. The effects of weight on fertility have been previously examined in broiler breeders but not in layer breeders. But although these studies report the impact of weight on semen parameters, some authors argue that its greater influence comes from reduced sexual activity due to behavioral, hormonal, or even body conformation factors (Sarabia Fragoso et al., 2013; Oviedo-Rondón and Romero-Sanchez, 2022). Besides, there is still much debate within broiler lines regarding the weight ranges that yield the poorest fertility outcomes, since both overweight and underweight males face problems with testicular regression, body conformation, and mating performance. However, once again, there is no scientific literature discussing the role of the body weight on seminal parameters, sexual behavior or fertility in layer breeders, making it difficult to compare our data with others.

The fleshing score is an additional measure of critical importance in the management of broiler breeders due to its correlation with body weight and conformation, tarsus length, physical activity, and mating efficiency (Wolanski et al., 2004; Abad and Jarquin, 2022; Oviedo-Rondón and Romero-Sanchez, 2022). This index is considered relevant because, although roosters within the same flock may have similar weights, they do not exhibit the same body frame, fat reserves, or pectoral muscle mass, which in practice differentiate them in terms of reproductive performance (Wolanski et al., 2004; Abad and Jarquin, 2022; Oviedo-Rondón and Romero-Sanchez, 2022). For example, males with very high fleshing scores may be unable to maintain balance and complete copulations, whereas individuals with very low scores do not have access to females or lack the necessary reserves to remain adequate libido or good semen production. However, males from layer lines are typically more active and agile, not exhibiting the same profiles of abrupt reduction in libido nor the same aptitude for accumulating fat as their broiler counterparts. This can be clearly perceived when comparing fleshing scales used by broiler and layer breeding farms. Here, no differences were found among the three fleshing scores regarding TPSs, although we noticed a slight tendency for roosters with high fleshing scores to have higher TPSs. Considering that layer males do not tend to accumulate large fat reserves, we assume that individuals capable of doing so during their productive cycle may keep better levels of activity, libido and sperm production. Again, it is likely that very uneven sample sizes across fleshing score categories limited the statistical power of our analyses, making it difficult to find real population differences. Yet, since our study was designed to be a monitoring of field conditions rather than an experiment with individuals controlled within pre-established ranges, we anticipated that such asymmetries might occur. It is also worth mentioning that although TPS is a critical component of the male’s reproductive capacity, it does not necessarily represent fertility itself. Fertility is a multifactorial parameter that involves not only the males’ seminal potential but also their mating and copulation efficiency, social dominance hierarchies, male-to-female-ratios, housing densities, among other factors.

From a practical standpoint, an interesting data was the high percentage of underweight males showing both inactive cloacas and failures in semen collection. This situation suggests that, despite underweight breeders did not show significant differences in TPSs, they may not be in satisfactory condition to engage in hierarchical disputes with other males or possess sufficient libido to engage copulation. It is worth noting that in many of these cases the individuals that released ejaculates after digital massage did not demonstrate a well-developed response to the stimuli applied by the collector, meaning they produced semen but with little or no excitatory response (tail reflex or phallic engorgement). Of course, we cannot rule out the possibility that these roosters did not adapt to the semen collection procedure, but this fact combined with signs of cloacal inactivity led us to raise this hypothesis. This finding aligns with previous arguments in broiler breeders that decreases in fertility often arise from problems not necessarily linked to spermatogenic activity, but rather from steroidogenic or behavioral issues. Our assumption is further supported by the confirmation that males with low fleshing scores also had higher percentages of inactive cloacas, indicating that individuals with less muscle mass and fat accumulation may have expressed reduced libido. These results highlight the applicability of cloacal inspection in the selection routines of breeding males from layer lines. Likewise, we argue that the use of digital massage for semen collection (not necessarily for the purpose of analyzing or using semen for artificial insemination) may serve as an additional tool for selecting males. Nevertheless, managers should take into account important factors such as staff experience, logistics, flock size, among others for its incorporation in the routine of parent, grandparent and great-grandparent stocks. Adopting these practices in the daily routine could potentially have positive repercussions on egg fertility, and consequently on flock profitability, by removing layer breeding roosters that would otherwise be classified as active during weight and fleshing assessments.

In summary, the data generated by this study suggest that caution should be exercised when selecting breeding roosters based solely on body weight and fleshing criteria when dealing with layer lines. Furthermore, it was possible to observe the relationship between these indicators and the presence of active and inactive cloacas, as well as the males’ ability to respond to sexual stimuli. We stress that this was an initial work on this theme, and that further research involving hormonal and behavioral aspects under field conditions is still needed to reach more definitive conclusions on the matter. However, our findings serve as a constructive challenge directed at professionals in the field and genetic companies to reevaluate the current selection parameters for layer roosters throughout the production cycle.

## Declaration of interest statement

The authors declare no conflicts of interest.

## Ethics declaration

This study was conducted in accordance with the ARRIVE (Animal Research: Reporting of In Vivo Experiments) guidelines. This study was approved by the Ethics Committee of the College of Veterinary Medicine and Animal Sciences at the São Paulo University. (Approval No. CEUAVET No 5477171122)

## Author contributions

Eder B. Oliveira was involved in the conceptualization of the study, data collection in the parent house, supervision of the company's staff during collection, formal analysis and in the writing of the original draft and the revised version; Edvaldo F. S. Fogaça participated in data collection in the parent house, was responsible for the animal care and feeding throughout the experiments, provide data regarding roosters' physical characteristics, and was involved in the preparation of the original draft; Alan M. Durigon was responsible for the supervision of the parent house staff, provided funds and conditions for the development of the experiments at the partner company, was involved in sample collection, and helped in the preparation of the original draft and the revised versiont; Marcílio Nichi was involved in the experimental design and formal analysis, in the writing the manuscript draft, and reviewing final version of the manuscript. Ricardo Jose Garcia Pereira designed the experimental processes, was involved in sample collection and formal analysis, coordinated the collaboration between lab and parent house staffs, provided the funds to support this research. He was also responsible for the preparation of the original draft and the revised version. Ricardo.

## Data Availability

The datasets generated during the current study are available from the corresponding authors on reasonable request.
